# Clinical characterisation of human metapneumovirus infection in 525 patients with respiratory tract infections in Jilin province

**DOI:** 10.3389/fcimb.2025.1616548

**Published:** 2025-08-20

**Authors:** Xiuhua Liu, Yang Liu, Jing Bian, Mengjuan Wang, Zhanxiu He, Huan Hu, Lei Zhang, Chaoying Liu

**Affiliations:** ^1^ Department of Respiratory Medicine, The First Hospital of Jilin University, Changchun, China; ^2^ Zhejiang Key Laboratory of Digital Technology in Medical Diagnostics, Medical Research Center, Hangzhou, China; ^3^ Clinical Biological Sample Center, The First Affiliated Hospital of Jinzhou Medical University, Jinzhou, China

**Keywords:** human metapneumovirus, epidemiology, targeted next generation sequencing, tNGS, lower respiratory tract infection, viral infection

## Abstract

**Background:**

Human metapneumovirus (HMPV) is a common cause of acute respiratory infections. The aim of this study was to analyze the demographic characteristics and treatment outcomes of HMPV virus infection in Jilin province.

**Methods:**

We conducted a retrospective cohort analysis of patients with respiratory tract infections between September 2023 and February 2024 in the Lequn Campus of the First Hospital of Jilin University, using tNGS sequencing. This study focused on HMPV-infected patients and included infections with this virus alone as well as co-infections.

**Results:**

In the present study, 525 patients with respiratory diseases were analysed, 65 (12.57%) of whom were found to be infected with HMPV. The period of maximum human metapneumovirus infection was observed to be January, and of the 65 patients infected with this virus, 10 (15.2%) were infected with HMPV alone and 56 (84.8%) were co-infected with HMPV. The most prevalent co-infection was bacterial, with the most common identified pathogen being human herpesvirus, followed by Candida albicans and Streptococcus pneumoniae. The percentage of co-infections in patients not infected with HMPV was 78.87%. Patients infected with HMPV alone exhibited a lower proportion of males, elevated rates of fever and cough, and a higher prevalence of diabetes mellitus and CURB-65 score (P>0.05). The most prevalent initial diagnoses were pneumonia, with additional respiratory failure and hypoproteinaemia diagnoses. Furthermore, a higher proportion of patients infected with the HMPV virus were hospitalised for more than 10 days compared with those not infected with the virus.

**Conclusion:**

HMPV is easily neglected in the current diagnosis and treatment process, but the risk it poses, such as long hospitalisation, should not be ignored. The tNGS showed excellent detection performance and great potential in this study, and it can be a good tool to help clinicians in diagnosis and treatment.

## Introduction

Human metapneumovirus (HMPV), initially discovered by Van den Hoogen in 2001 (van den Hoogen et al., 2001), is a globally prevalent respiratory virus capable of infecting individuals across all age groups (Ogonczyk Makowska et al., 2020). However, it exhibits a higher prevalence among infants, young children, the elderly, and immunocompromised individuals. Several studies have indicated that HMPV can be the sole pathogen detected in patients with community-acquired pneumonia, suggesting its potential to cause disease independently (Nascimento-Carvalho et al., 2011; Del Rosal et al., 2024). HMPV is an RNA virus belonging to the family Paramyxoviridae, subfamily Pneumoviridae, and genus Metapneumovirus, sharing a distant genetic relationship with Respiratory Syncytial Virus (RSV), Parainfluenza Virus, Measles Virus, and Mumps Virus (Milder and Arnold, 2009).

In recent years, there has been a notable increase in HMPV infections globally. Since the first documented outbreak in China in 2003 (Chan et al., 2003), the virus has garnered significant attention due to its potential public health implications. The CDC has reported a continuous rise in acute respiratory infectious diseases, with a particularly evident increase in influenza cases and a gradual rise in HMPV infections. In 2023, the United States experienced a large-scale epidemic of HMPV. According to the U.S. Centers for Disease Control and Prevention’s (CDC) respiratory virus surveillance system, HMPV has shown high prevalence across various regions since spring 2023, impacting intensive care units and pediatric wards in major hospitals. Over 19% of PCR tests and more than 10% of antigen tests from respiratory secretion samples have been positive for HMPV. Individuals infected with HMPV are contagious from the end of the incubation period through the acute phase, and the duration of immunoprotection post-infection is relatively short, allowing for the possibility of repeated infections. Human metapneumovirus (HMPV) and respiratory syncytial virus (RSV) are both recognised causes of infections affecting both the upper and lower respiratory tracts (Nadiger et al., 2023). HMPV infections exhibit a distinct seasonal pattern, circulating year-round with a notable increase in prevalence during the late winter and spring months. This peak often coincides with, or follows shortly after, the peak incidence of respiratory syncytial virus (RSV) (Williams et al., 2004).

Symptoms of HMPV infection typically manifest as upper respiratory tract infection symptoms, including fever, cough, nasal congestion, runny nose, and hoarseness, with most symptoms resolving within approximately one week. In severe cases, complications such as capillary bronchiolitis, severe pneumonia, and acute respiratory distress syndrome (ARDS) may develop. HMPV infection can cause severe bronchitis and pneumonia in children, with symptoms similar to those caused by the human respiratory syncytial virus (RSV). The initial hMPV infection usually occurs in early childhood, but reinfection is common throughout life (Panda et al., 2014). Statistics on severe pneumonia in hospitalised children over a two-year period in seven African and Asian countries showed that respiratory syncytial virus (RSV) was found in 31.1% of cases, rhinovirus in 7.5%, and human metapneumovirus (HMPV) in 7.5%. HMPV was therefore the third most common cause of severe pneumonia in children (Group PERfCHPS, 2019).

Currently, there is no vaccine or specific treatment regimen available for pneumonia caused by HMPV infection (Guo et al., [[NoYear]]). Severely affected individuals commonly include children under the age of five, the elderly, and patients with underlying cardiac, pulmonary, or immunocompromised conditions (Martinez-Rodriguez and Banos-Lara, 2020). HMPV is increasingly being associated with chronic lung transplant dysfunction (CLAD) in lung transplant recipients (LTRs) (de Zwart et al., 2022). Treatment strategies for these patients primarily involve supportive care and steroid-enhanced therapy. There are limited antiviral treatment options, such as ribavirin (RBV), palivizumab or intravenous immunoglobulin (IVIG). The broad-spectrum nucleoside analogue RBV exhibits *in vitro* activity against HMPV (Wyde et al., 2003; Smielewska et al., 2018). Various new antiviral drugs, including interfering RNAs and fusion proteins, are being tested for the treatment of these infections (Verleden et al., 2019).

## Materials and methods

### Study population and data collection

This retrospective observational study focused on determining the prevalence of human metapneumovirus (HMPV) among adult patients (aged ≥18 years) hospitalised with a diagnosis of lower respiratory tract infection. The study encompassed patients admitted to the First Hospital of Jilin University, spanning the period from September 2023 to February 2024. Data collected included demographics, primary medical conditions, symptoms of infection, time from onset to admission, laboratory test results, duration of illness, length of hospital stay, complications, and recovery period. The Confusion-Urea-Respiratory rate-Blood pressure-Age 65 (CURB-65) score was calculated for each patient to assess the severity of the disease.

Ethical approval for the study was obtained from the Ethics Committee (registration number: 2025-70), ensuring adherence to ethical guidelines. Patient anonymity was rigorously maintained throughout the study. Informed consent was waived, given the retrospective and observational nature of the study and its critical role in the public health response, particularly in the context of an emerging health concern.

### Sample collection

Bronchoalveolar lavage fluid (BALF) or sputum samples were meticulously collected from eligible patients and subsequently stored in sterile screw-cap freezer bottles. It is noteworthy that BALF samples were specifically procured from the mid-segment, with fluid obtained from the anterior segment being systematically discarded. In a similar vein, sputum samples were carefully collected from the patient’s first deep cough in the early morning and were rinsed 2–3 times with sterile saline to ensure purity. These samples were promptly transported to a designated laboratory for targeted next-generation sequencing (tNGS) at a temperature of ≤ -20°C to preserve sample integrity. For BALF samples, a volume of 5–10 mL was carefully collected from each patient, while 4 mL of sputum was collected per patient. These samples were processed and preserved with utmost care to maintain the quality of the genetic material, thereby ensuring the reliability and accuracy of the subsequent tNGS analysis.

### Targeted next-generation sequencing

Sputum and viscous alveolar lavage fluid (BALF) were added to the working solution of the digestive solution and briefly vortexed and shaken until the specimen was liquefied. The BALF liquid or thickened sputum was then mixed with DNA/RNA Shield and placed into a wall-breaking apparatus to disrupt cellular structures. Subsequently, the sample was subjected to gentle centrifugation, and the supernatant was collected for subsequent DNA extraction. Nucleic acid extraction or purification reagents were added to the supernatant, and DNA/RNA co-extraction was performed using a fully automated nucleic acid extractor.

For the purpose of library preparation, the Genseq RTI Panel library construction kit was utilised for the preparation of DNA libraries and cDNA libraries. The products were then purified using magnetic beads, after which the concentration of the purified products was determined and recorded using a Qubit 4.0 Fluorometer. Basic information regarding the prepared libraries was entered into the mixing operation table, and the “concentration measured in the mixing pool” was calculated. It is imperative to ensure the accuracy of experimental results; therefore, the difference between the measured concentration of the mixed sample and the theoretical concentration should be maintained within 15%.

Following the preparation of mixed samples into DNA nanoballs (DNBs), they were subjected to sequencing on a MGI-200 gene sequencer. Subsequent to this, the pathogenic microorganism sequencing data analysis software Genseq-PM automatically recognised the downstream data, performed quality control on the raw data, and compared it with an independently constructed pathogen database for species identification.

### Statistical analysis

Discrete variables were characterised using frequencies and percentages, whereas continuous variables were described by the median and interquartile range (IQR). The chi-square test was used to conduct comparisons of discrete variables. For continuous variables, the Mann-Whitney U test was employed for comparisons. The organisation, analysis and visualisation of the data was facilitated using the R programming language, with the rstatix, ggplot2, zoo and ggpubr packages being utilised, amongst others. The data were analysed using the Statistical Package for the Social Sciences (SPSS) version 26.0 (IBM Corporation, Armonk, NY). A p-value threshold of <0.05 was established in order to determine statistical significance.

## Results

**Figure 1:**
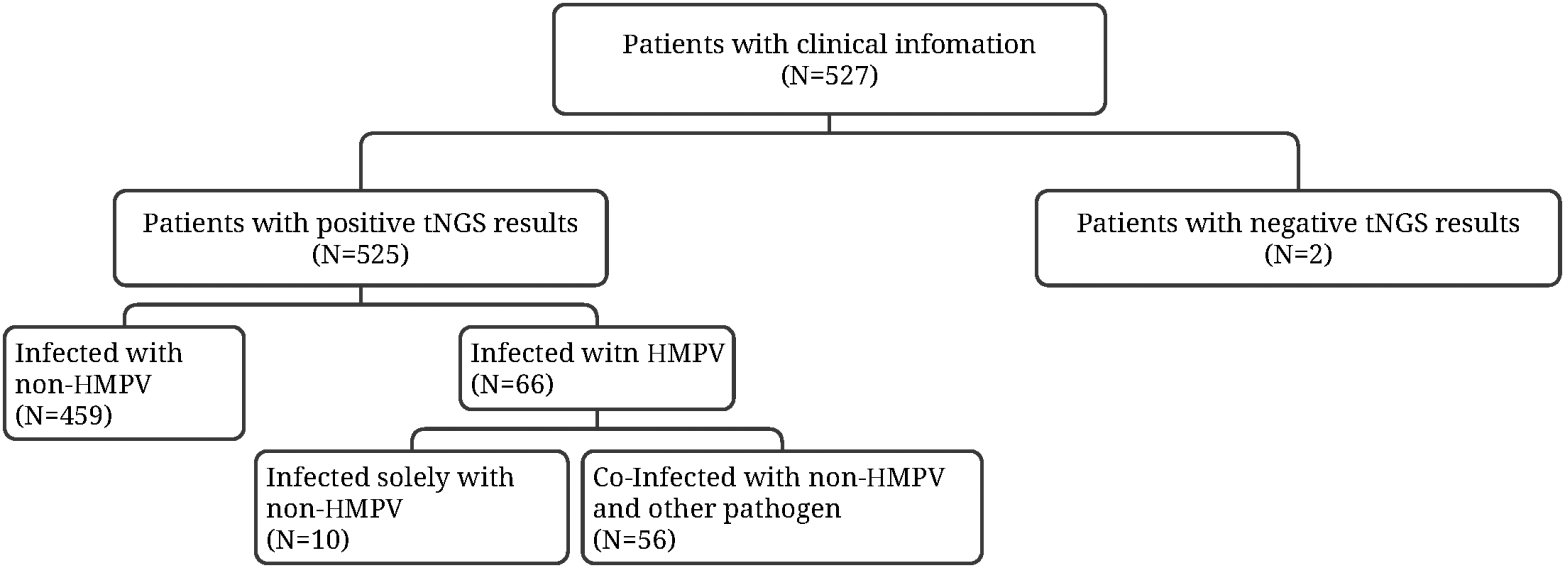
Subgroup of patients with lower respiratory tract infections.

The study enrolled 527 patients with lower respiratory tract infections for preliminary testing between September 2023 and February 2024. Two patients exhibited a negative result. The final study population comprised 525 patients. The 525 patients underwent tNGS, which detected positive results. Specifically, 10 patients (15.2%) were found to have HMPV infection alone, while 56 patients (84.8%) had dual infection with HMPV and other pathogens. The remaining 459 patients were not infected with HMPV (**Figure 1**).

The demographic and clinical characteristics of patients infected with HMPV and those uninfected with the virus in the study population are detailed in **Table 1**. Patients infected with HMPV exhibited distinct demographic characteristics compared to those uninfected. Specifically, the infected group had a higher proportion of males and a higher proportion of concomitant cough and fever characteristics. Furthermore, the infected group exhibited a higher proportion of concomitant hypertension, but a lower proportion of diabetes, and a lower proportion with a history of smoking and alcohol consumption, based on the patients’ concomitant diseases. In the context of asthma and tuberculosis, the proportions of the infected and non-infected HMPV groups were found to be nearly equivalent.

**Table 1:** Demographic and clinical characteristics according to the presence or absence of HMPV infection.

Characteristics	Patients infected with non-HMPV (N=459)	Patients infected with HMPV (N=66)	*P*-value
Male, n (%)	241 (53)	38 (58)	0.5222
Age (years), median (IQR)	66 (53–73)	65 (54–73)	0.5983
Smoking, n (%)	167 (36)	22 (33)	0.7849
Drinking, n (%)	74 (16)	5 (8)	0.11
Cough, n (%)	422 (92)	63 (95)	0.4482
Fever, n (%)	254 (55)	41 (62)	0.365
Hypertension, n (%)	128 (28)	21 (32)	0.6056
Diabetes, n (%)	90 (20)	11 (17)	0.6893
Asthma, n (%)	7 (2)	1 (2)	1
Pulmonary tuberculosis, n (%)	23 (5)	4 (6)	0.9498
From onset to admission (days), median (IQR^a^)	5(0-11.5)	5 (3–10)	< 2.2e-16

a IQR, interquartile range.

A number of clinical distinctions were identified between patients with HMPV infection alone and those with co-infection. Patients with HMPV infection alone were less likely to be male. With respect to morbidity features, the proportion of fever and cough was higher. The prevalence of underlying diseases was higher for diabetes mellitus and lower for hypertension, asthma and tuberculosis. Patients with HMPV infection alone exhibited a low prevalence of smoking, yet a high prevalence of alcohol consumption. Furthermore, patients with HMPV infection alone had a longer time from onset to admission (**Table 2**).

**Table 2:** Demographic and clinical characteristics of monoinfection with HMPV and multipathogen infection.

Characteristics	Patients only infected HMPV (N=10)	Multiple pathogens(N=55)	P-value
Male, n (%)	5 (50)	33 (60)	0.858
Age (years), median (IQR)	67 (53-73.5)	64.5 (54–73)	0.4934
Smoking, n (%)	3 (30)	19 (35)	1
Drinking, n (%)	1 (10)	4 (7)	1
Cough, n (%)	10 (100)	53 (96)	1
Fever, n (%)	8 (80)	33 (60)	0.3621
Hypertension, n (%)	1 (10)	20 (36)	0.2151
Diabetes, n (%)	2 (20)	9 (16)	1
Asthma, n (%)	0 (0)	1 (2)	/
Pulmonary tuberculosis, n (%)	0 (0)	4 (7)	/
From onset to admission (days), median (IQR)	5 (4.25-7)	4 (2.75-10)	0.09296

As demonstrated in **Table 3**, there were significant differences in the primary clinical tests between patients categorised according to whether they were infected with HMPV. The proportion of uninfected patients with a CURB-65 score greater than or equal to 2 was higher (81%), and the proportion of patients infected with HMPV was only 13%, which was significantly different (*p*<0.05). A subsequent investigation into the results of routine blood tests revealed that there was no statistically significant difference between the two groups (*p* > 0.05). A comparative analysis was conducted on the characteristics exhibited by patients infected with the HMPV virus in isolation and those who were co-infected(**Table 4**). The findings revealed that there were no statistically significant differences (*p* > 0.05) in the common blood test results and CURB-65 scores between the two groups.

**Table 3:** Clinical examination at admission according to the presence or absence of HMPV infection.

Examination	Patients infected with non-HMPV (N=459)	Patients infected with HMPV (N=66)	*P*-value
CRP (mg/L), median (IQR)	43.27 (10.75-99.46)	50.50 (8.69-108.34)	0.91
PCT (mg/L), median (IQR)	0.11 (0.05-0.26)	0.13 (0.05-0.22)	0.87
White blood cell count, median (IQR)	8.21 (5.81-11.24)	7.09 (4.98-10.72)	0.29
Neutrophil ratio, median (IQR)	76.90 (63.92-86.58)	79.00 (67.25-88.00)	0.69
Lymphocyte ratio, median (IQR)	14.00 (7.60-23.07)	14.20 (7.15-21.40)	0.63
Fungal D-glucan, median (IQR)	37.50 (37.50-40.533)	37.50 (37.50-43.18)	0.93
galactomannan, median (IQR)	0.08 (0.06-0.13)	0.09 (0.07-0.16)	0.10
CURB-65 score ≥ 2, n (%)	81 (0.18)	13 (0.2)	0.03

CRP, C-reactive protein; PCT, procalcitonin; CURB-65, confusion urea respiratory rate blood pressure age 65.

**Table 4:** Clinical examination at admission of monoinfection with HMPV and multipathogen infection.

Examination	Patients only infected HMPV (N=10)	Multiple pathogens (N=55)	*P*-value
CRP (mg/L), median (IQR)	83.5 (67–100)	41.60 (7.77-103.07)	0.49
PCT (mg/L), median (IQR)	0.70 (0.39-1.01)	0.13 (0.05-0.20)	0.38
White blood cell count, median (IQR)	10.18 (9.96-10.39)	6.73 (4.96-10.82)	0.33
Neutrophil ratio, median (IQR)	90.55 (89.97-91.12)	74.40 (65.60-86.60)	0.09
Lymphocyte ratio, median (IQR)	6.50 (6.30-6.70)	15.50 (7.80-21.60)	0.18
Fungal D-glucan, median (IQR)	37.50 (37.50-37.50)	37.50 (37.50-46.62)	0.39
Galactomannan, median (IQR)	0.07 (0.07-0.07)	0.09 (0.07-0.16)	0.25
CURB-65 score ≥ 2, n (%)	4 (0.40)	9 (0.16)	0.20

**Figure 2:**
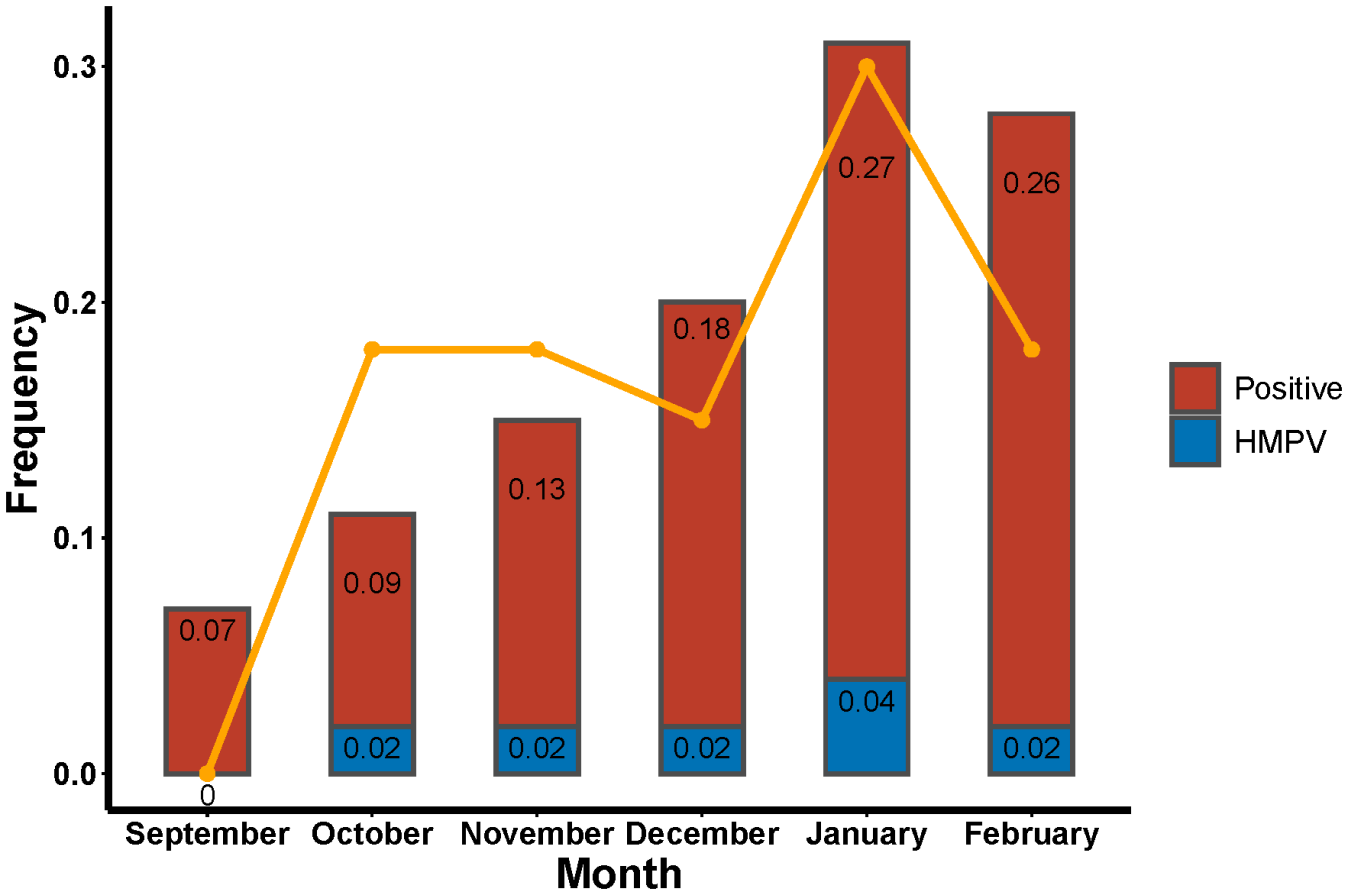
Rates of tNGS and HMPV positivity in patients with lower respiratory tract infections and proportion of HMPV positivity in a single month.

The number of patients diagnosed with lower respiratory tract infections exhibited an upward trend from September 2023 to February 2024. A decline in patient numbers was observed in September and October, with these patients constituting 6.86% and 9.14% of the total study population, respectively. Conversely, an increase in patient numbers was observed in January and February 2024, with these patients constituting 27.05% and 25.90% of the total study population, respectively. The prevalence of HMPV infections was observed to occur in October (18.18%), November (18.18%), December (15.15%), January (30.30%) and February (18.18%). The highest positivity rate was observed in January (**Figure 2**). The data is presented using two graphical representations: a bar graph, which illustrates the number of infections compared to the total study population, and a line graph, which compares the number of HMPV infections in a given month to the total number of HMPV infections for that period.

**Figure 3:**
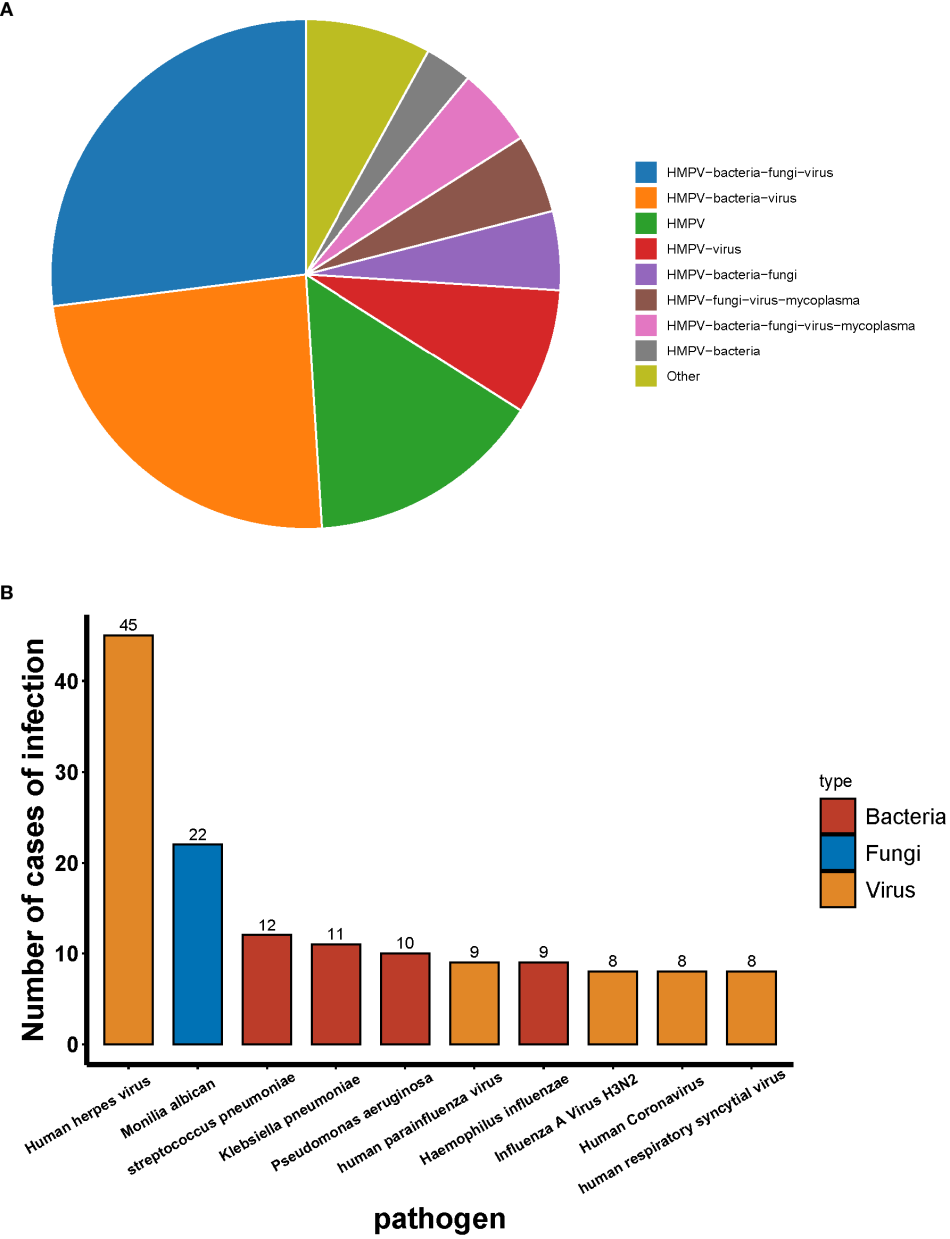
Distribution of infection types in patients infected with HMPV.

A total of 55 patients were infected with HMPV, of whom 15.15% were infected with HMPV alone and 84.45% were co-infected with other pathogens. The most prevalent types of co-infection included: co-infection with bacteria and viruses (24.24%), co-infection with bacterial and fungal and viruses (27.27%), and the remaining types of co-infection were less than 10% (**Figure 3A**). With the exception of HMPV, the most prevalent pathogen detected by tNGS was the human herpes virus, followed by Candida albicans and Streptococcus pneumoniae (**Figure 3B**).

**Figure 4:**
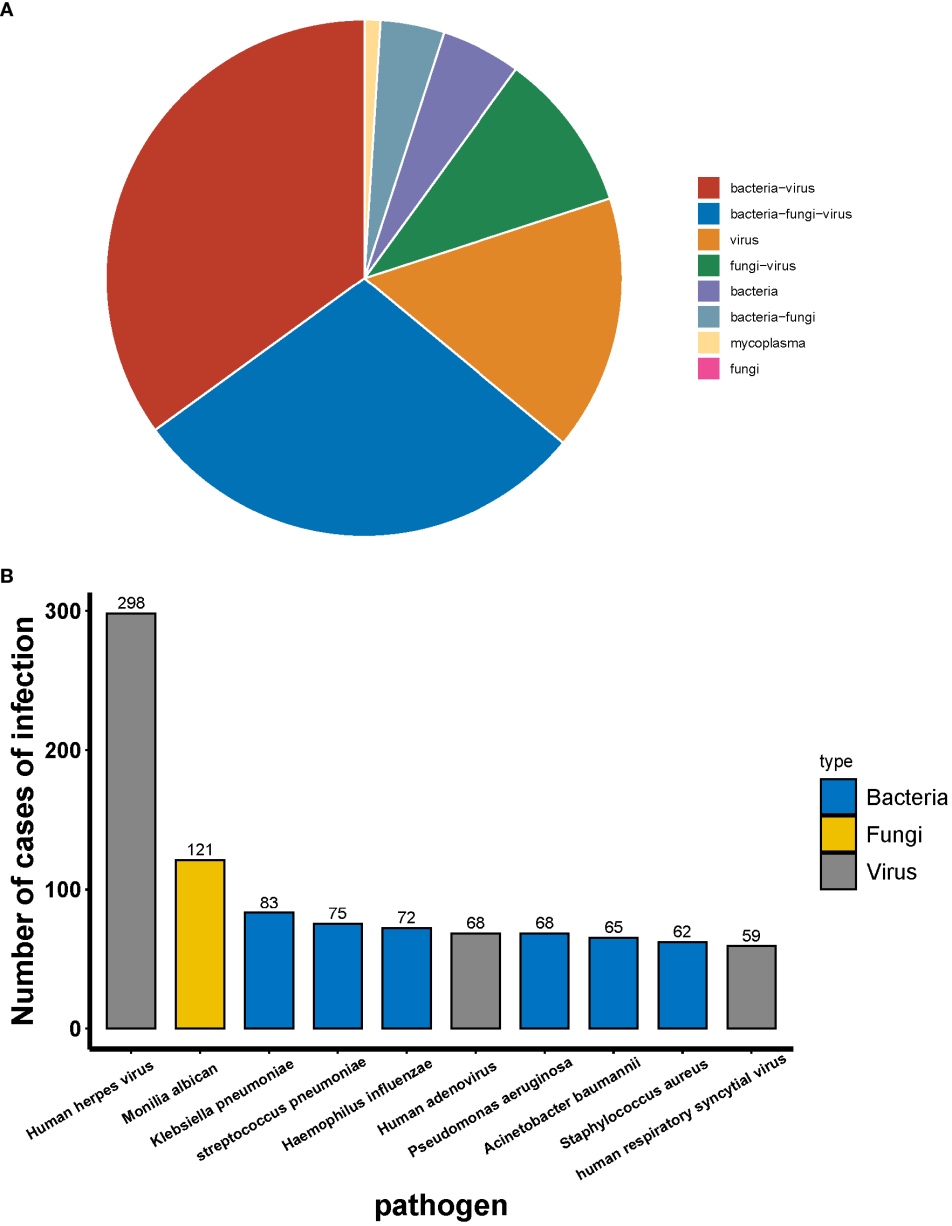
Distribution of infectious agents in patients infected with HMPV.

Among the 459 patients who were not infected with HMPV, the most common type of infection was co-infection (78.87%) and single infection (21.13%). The most prevalent co-infecting pathogens were bacteria and viruses (34.86%) and bacteria, fungi and viruses (28.76%) (**Figure 4A**). Among the co-infections, tNGS identified human herpesvirus as the most prevalent pathogen, followed by Candida albicans, Klebsiella pneumoniae and Streptococcus pneumoniae (**Figure 4B**).

Through the meticulous enumeration of data pertaining to viral infections in the enrolled patients, it was ascertained that human metapneumovirus exhibited the most pronounced correlation with rhinoviruses, evidenced by a correlation coefficient of 0.21. Furthermore, HMPV demonstrated a negative correlation with the preponderance of DNA viruses, while concurrently exhibiting a positive correlation with the majority of RNA virus infections (**Table 5**).

**Table 5:** Correlation coefficient for viral co-infections.

Name of the virus	Type of virus	Correlation
Human adenovirus	DNA	-0.04
Human Herpesvirus	DNA	-0.02
Enterovirus C	RNA	-0.02
Human Bocavirus	DNA	-0.02
Human minuscule virus	DNA	-0.02
Human Respiratory Syncytial Virus	RNA	-0.01
Coronavirus	RNA	0.00
Influenza A virus H3N2	RNA	0.01
Influenza B virus	RNA	0.01
Human Parainfluenza Virus	RNA	0.06
Rhinovirus	RNA	0.21

A meticulous examination of the initial diagnoses of patients afflicted with lower respiratory tract infections reveals that pneumonia was the most prevalent diagnosis, with a total of 501 cases, constituting 95.4% of the total. This is accompanied by other notable diagnoses, including hypoproteinaemia, anaemia, respiratory failure, and liver injury (**Table 6**). The statistical analysis of the duration of hospitalisation revealed that patients infected with HMPV alone constituted the highest proportion of hospitalised patients, with 50.0% of cases requiring hospitalisation for more than 10 days. Furthermore, the proportion of patients infected with HMPV who were hospitalised for more than 10 days was higher in the group of patients infected with HMPV (42.4%) compared to patients not infected with HMPV (26.8%) (**Table 6**).

**Table 6:** Statistics on the results of initial diagnosis and length of hospitalisation.

Characteristics	Patients infected with non-HMPV (N=459)	Patients infected with HMPV (N=66)	Patients only infected HMPV (N=10)	Multiple pathogens (N=55)
Respiratory failure	123	19	3	16
Liver damage	108	18	2	16
Hypoproteinaemia	131	20	3	17
Electrolyte disorders - Hypokalemia	104	20	3	17
Hypoxemia	81	12	1	11
Renal Insufficiency	68	8	1	7
Renal cysts	51	8	2	6
Pneumonia	440	61	10	51
Anaemia	102	21	5	16
Hospitalisation for more than 10 days	123 (26.8%)	28 (42.4%)	5 (50.0%)	23 (41.82%)

## Discussion

In this study, the spectrum of viral and bacterial pathogens was determined, and their differences in patient demographics were explored, by using surveillance data from patients with acute respiratory infections in Jilin Province. A total of 525 patients were tested positive for tNGS in this study, and 66 patients were infected with HMPV, accounting for 12.57% (66/525) (**Figure 1**). A study on HMPV infection in children’s respiratory tract revealed that HMPV was detected in 524 (3.5%) children, with the majority being less than 1 year of age (Ye et al., 2023). A study by Aberle et al. further demonstrated that 202 out of 3,576 samples (5.6%) were positive for HMPV (Aberle et al., 2010). A further study of HMPV infection in China involved the analysis of a total of 188,104 clinical samples, of which 8846 were positive for HMPV, yielding a mean positivity rate of 4.70% (Feng et al., 2024). As the seasonal peak of HMPV prevalence is primarily observed in the spring (Group PERfCHPS, 2019), the period of sample collection corresponded to the inclusion of winter and spring, which may have contributed to the high percentage of positive HMPV infection observed in this study. The prevalence of viral infection was found to be associated with the region of the patient, and the prevalence of HMPV in patients with acute respiratory diseases varied significantly among different regions of China, with the percentage of positivity ranging from 0.97% to 15.88%.The highest prevalence of HMPV was observed in Chongqing and Henan provinces, while the lowest prevalence rates were found in Shanghai, Jiangxi, Hainan, and Beijing, which accounted for less than 3.0% of the total (Feng et al., 2024). A six-year, single-centre, prospective study was conducted, observing a semiannual cycle of HMPV, with alternating peaks in winter and early spring. This cycle was hypothesised to be due to strain mutation and herd immunity from the previous season (Davis et al., 2015). This study has drawn clinicians’ attention to the HMPV infection in Northeast China and has provided inspiration and data support for future studies.

The findings of this study demonstrated that the number of patients with lower respiratory tract infections exhibited a progressive increase from September to January, with a decline in infections from January to February. Conversely, the HMPV infection rate reached its peak in January and February, accounting for 27% and 26% of all infected patients in those months (**Figure 2**). This trend aligns with the findings of previous studies. A study conducted in multiple provinces and cities in China further corroborates this finding, demonstrating that patients exhibited the highest positive rate of HMPV infection in the spring (12.13%), followed by winter (3.56%), summer (1.28%), and fall (1.32%) (Feng et al., 2024). The temporal variation in the prevalence of HMPV infection is evident across different geographical regions. In European and North American countries, the incidence of HMPV-positive cases is observed to be higher during the winter months, while in some Asian countries, a peak in infections has been recorded during the rainy season (Williams et al., 2006; Aberle et al., 2010). A study of respiratory infections in the United States demonstrated that HMPV positivity reached its zenith in April. From 4 January 2020 to 14 March 2020, the weekly percentage of HMPV positive results increased from 4.2% to 7.0%, declined to 1.9% during the week of 11 April 2020, and remained at less than 1.0% through to 22 May 2021. Over the past four years, HMPV circulation has peaked in March and April at 6.2% to 7.7% (Olsen et al., 2021).

In this study, it was found that the proportion of males was higher in patients infected with HMPV, and the proportion of patients with accompanying cough and fever features was higher compared to patients not infected with HMPV. However, the difference was not significant (*P* > 0.05) (**Table 1**).

Studies have reported the detection of HMPV infection in certain patients, thereby suggesting that HMPV is capable of causing disease in isolation (Nascimento-Carvalho et al., 2011; Del Rosal et al., 2024). The present study’s cohort comprised 10 patients infected with HMPV alone and 56 patients who were co-infected with HMPV and other pathogens (**Figure 1**). With regard to gender distribution, a lower proportion of males was observed among patients infected with HMPV alone. However, the small number of patients infected with HMPV alone (n = 10) meant that the ratio of men to women was affected by the scope of collection, potentially introducing some bias.

Patients infected with HMPV alone and those co-infected with other pathogens exhibited discrepancies in clinical features and general basic information. With respect to morbidity characteristics, the proportion of fever and cough was higher in patients infected with HMPV alone. In terms of underlying diseases, the prevalence of diabetes was higher, and the statistical results of lifestyle habits showed that the proportion of alcohol consumption was higher in patients infected with HMPV alone. Furthermore, the data on the time of visit revealed that patients infected with HMPV alone had a longer time from the onset of the disease to admission to the hospital. Patients infected with multiple pathogens presented to the hospital earlier than those infected alone, and it can be hypothesised that mixed infections with multiple pathogens can exacerbate the severity of the disease. However, the fever and cough were more prevalent in patients infected with HMPV alone, which also supports the hypothesis that the virus is more pathogenic. Studies have demonstrated a growing body of evidence that supports the use of basic clinical data in the early prediction of influenza-related diseases. This development has the potential to facilitate rapid diagnostic and treatment decisions, leading to improved patient care and a reduction in the duration of hospitalisation.

The findings of this study demonstrated that among the 55 patients who were co-infected with HMPV, a mere 15.15% were found to be infected with a single type of HMPV virus. Human respiratory syncytial virus (HRSV) has been shown to be the virus most commonly co-infected with human metapneumovirus (HMPV) (5.3%) (Ye et al., 2023).HMPV infection has been observed to be synchronised with or shortly after HRSV (Williams et al., 2004).The results of the present study demonstrated a weak correlation between HMPV and RSV infections, which may be attributable to the fact that the patients were already infected with RSV in the preexisting period, which was not detected in the present test. The findings of viral coinfection may be regionally related. Madewell (Madewell et al., 2023) enumerated the interactions of acute respiratory viruses in four Chinese cities from 2009-2019, encompassing Beijing, Chongqing, Guangzhou, and Shanghai. The study demonstrated that there was also a substantial degree of spatial heterogeneity in virus-virus interactions, such as between IAV and HRV, and HMPV, and between RSV-A and RSV-B, which was only observed in Chongqing (Madewell et al., 2023). Chongqing’s climatic conditions are typified by a subtropical monsoon climate, characterised by high relative humidity. Previous studies have demonstrated a correlation between meteorological factors in Chongqing and the prevalence of influenza and pertussis (Wang Y. et al., 2020; Qi et al., 2021). Consequently, this study is instrumental in elucidating the infection trend in northeast China during the winter and spring months. Moreover, if other pathogens with a strong association with HMPV are identified, greater emphasis can be directed towards mitigating the progression of the disease.

A plethora of studies have demonstrated that the majority of statistically significant correlations among respiratory viruses are positive, a phenomenon that may be attributable to a multitude of factors, including shared transmission routes, interacting immune systems with the host, or environmental conditions that favour multiple virus types (Rouse and Sehrawat, 2010; Zhang et al., 2023). Furthermore, it is plausible that variations in transmission drivers shared by multiple viruses over time, such as unusual climatic conditions, patterns of social contact, or changes in healthcare-seeking behaviours, could result in similar deviations in the incidence of multiple viruses from their anticipated seasonal periodicity. This synchronisation bias could have contributed to the observed positive correlations between virus pairs. However, in this study, human-biased pneumovirus, an RNA virus, exhibited a positive correlation with most RNA viruses and a negative correlation with most DNA viruses. The underlying reason for this correlation remains to be elucidated and necessitates further scientific investigation.

HMPV is a significant pathogen that causes respiratory infections in children and adults (Haas et al., 2013; Feng et al., 2014). It has been determined that HMPV is among the ten most prevalent viruses in samples from paediatric oncology patients, with some cases of HMPV infection in cancer patients proving fatal (Martinez-Rodriguez and Banos-Lara, 2020). In susceptible individuals with underlying disease, HMPV infection can be fatal (Hermos et al., 2009). A Global Burden Assessment of Acute Lower Respiratory Tract Infections Associated with HMPV study, published in The Lancet Global Health in 2021, showed that in 2018, approximately 14.2 million cases of lower respiratory tract infections were associated with HMPV in children under 5 years of age globally, with approximately 643,000 of these requiring hospitalisation and more than 16,000 deaths, suggesting that HMPV can cause a significant disease burden (Wang X. et al., 2020). The 100-day mortality rate for HMPV infection in patients undergoing haematopoietic stem cell transplantation is approximately 43% (Renaud et al., 2013). Furthermore, the exacerbation of infection has been observed in patients diagnosed with chronic obstructive pulmonary disease (COPD), with the potential for acute exacerbations being induced in patients with bronchial asthma (Guo et al., [[NoYear]]). In the presence of immunocompromised patients, it is recommended that medical professionals prioritise HMPV testing. Obtaining a positive test result for HMPV should prompt the initiation of early and appropriate treatment, with the aim of controlling the patient’s condition and preventing its progression to a more severe state.

Broad-spectrum virotherapy drugs are often used to treat HMPV infection, and there is currently no targeted vaccine available. However, recent years have seen a significant advancement in the field, primarily due to breakthroughs in the structural biology of viral fusion proteins (F) and the accumulated knowledge from research with hRSV and other enveloped viruses. This has led to a marked acceleration in the development of Neutralising monoclonal antibodies (nMAbs) against HMPV. It is therefore reasonable to hypothesise that prophylactic and therapeutic vaccines against HMPV will enter the clinic in the future (Guo et al., [[NoYear]]).

## Conclusion

This study analysed the clinical characteristics of HMPV infection by comparing the results of HMPV-infected and uninfected individuals, and those infected with the virus alone and in combination with other pathogens. The results of the study are summarised in terms of the number of months with a high frequency of infection in patients, treatment outcomes, treatment duration and co-infections. This is to encourage more physicians to pay attention to HMPV viral infections and avoid delaying patients’ treatment. Early detection and treatment can shorten the treatment period for patients and reduce the suffering caused by the disease. Targeted treatment of infectious strains is a long-standing research topic for clinicians.

## Data Availability

The datasets presented in this study can be found in online repositories. The names of the repository/repositories and accession number(s) can be found in the article/supplementary material.
